# Fermented milk containing *Lactobacillus* GG alleviated DSS-induced colitis in mice and activated epidermal growth factor receptor and Akt signaling in intestinal epithelial cells

**DOI:** 10.3402/mehd.v23i0.18586

**Published:** 2012-06-18

**Authors:** Kazutoyo Yoda, Fang He, Kenji Miyazawa, Masaru Hiramatsu, Fang Yan

**Affiliations:** 1 Technical Research Laboratory, Takanashi Milk Products Co., Ltd., Yokohama, Kanagawa, Japan; 2 Department of Pediatrics, Division of Gastroenterology and Nutrition, Vanderbilt University School of Medicine, Nashville, USA

**Keywords:** Lactobacillus *GG*, fermented milk, colitis, EGF receptor, intestinal epithelial cell

## Abstract

*Lactobacillus rhamnosus* GG was assessed for its ability to alleviate DSS-induced colitis in mice and activate epidermal growth factor receptor and Akt signaling in intestinal epithelial cells. In this study mice were treated with DSS to induce colitis and they were given *Lactobacillus* GG fermented milk to assess the effect of probiotic on colitis. *Lactobacillus* GG fermented milk significantly reduced the colitis associated changes suggesting a protective effect against DSS induced colitis.

*Lactobacillus* GG (LGG, ATCC53103) is a widely used probiotic bacterium, which was originally characterized with strong adhesion to human intestinal mucus and epithelial cells. This bacterium can temporarily colonize the human colon following oral administration, and many health promoting effects have been documented during its persistence in the human intestine. Among these health benefits is its strain-dependent characterized ability to alleviate/improve the intestinal disorders of host animals such as antagonisms against various pathogenic bacteria and viruses associated with diarrhea, and inflammatory bowel disease (IBD). Therefore, additional knowledge about the underlying mechanism and key components related to these LGG actions will greatly help to characterize this probiotic bacterium much more effectively.

Recently, LGG was found to secrete two new functional soluble proteins, p40 and p75, in an artificial chemical culture medium (1). These functional proteins expressed apparent anti-inflammatory effects and regulated the survival and growth of epithelial cells in several cell culture and animal studies (2). Therefore, the production of these functional proteins might be one of the important requests for LGG to express its characterized health promoting effects to host intestinal function. However, it is still unclear if these functional proteins could be produced by LGG in the fermented milk, which is one of the most important food lines to use LGG as probiotics. The present study was conducted to demonstrate if these functional proteins could be detected from fermented milk product containing LGG (LGG-milk), and then investigate actions of LGG-milk on cultured epithelial cells and DSS-induced colitis in mice.

## Experimental

A total of 17 commercial fermented milks were tested for the possibility to secret p40 and p75 using Western-blotting analysis with anti-p40 and anti-p75 antibodies.

Young adult mouse colon (YAMC) cells were cultured with 1:100 to 1:2,000 diluted supernatant of LGG-milk for 2 h. Cellular lysates were prepared for Western-blotting analysis using anti-phospho EGF receptor-Tyr1068 and anti-phospho Akt antibodies to detect EGF receptor and Akt activation, respectively.

In order to evaluate the preventive effect of LGG-milk on DSS-induced colon epithelial injury and colitis, female C57BL/6 mice were treated with 3% DSS in drinking water for 4 days to induce colon injury and acute colitis. LGG-milk (500 µl) was gavaged to mice 6 days before and during DSS treatment. Inflammation and injury was assessed using a score system.

## Results

**Fig. 1:**
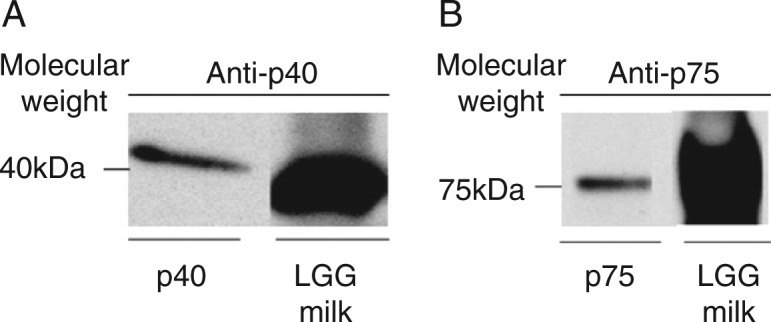
The functional proteins p40 (A) and p75 (B) identified in LGG-milk.

Fourteen fermented milk were found to contain p40 and p75, respectively. The highest concentration of p40 and p75 was found in the LGG-milk (Fig. 1). These results suggest that relative stronger ability to produce p40 and p75 might be one of the specific respects of LGG-milk.

**Fig. 2:**
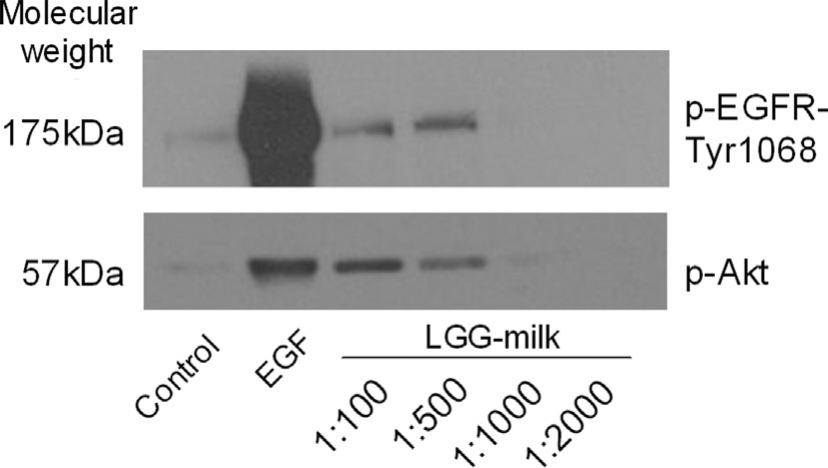
EGFR signal transduction activation with LGG milk.

LGG-milk activated EGF receptor and Akt in a concentration-dependent manner in the YAMC cells as same as those observed in the previous studies (Fig. 2).

**Fig. 3:**
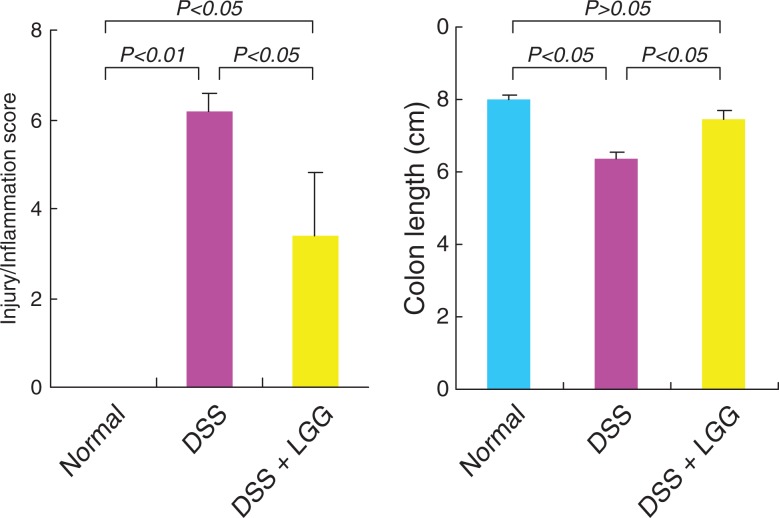
Epithelial damage in mice receiving DSS with or without LGG-milk.

The colitis (score: 6.2±0.84) was significantly lower following LGG-milk treatment (score: 3.4±3.14) compared to the other (Fig. 3). The shortening of the colon induced by DSS (6.2±0.39 cm), as a marker for colitis, was reduced by LGG-milk treatment (7.48±0.48 cm) (Fig. 3). These results suggest that LGG-milk may protect mice from DSS-induced colitis.

## Conclusion

These studies suggest that LGG can secrete functional soluble proteins, p40 and p75 in the fermented milk. These functional proteins could be considered as among the key components to determine the potent health promoting effects of LGG-milk to host animal.
